# Optimized design of antisense oligomers for targeted rRNA depletion

**DOI:** 10.1093/nar/gkaa1072

**Published:** 2020-11-22

**Authors:** Wesley A Phelps, Anne E Carlson, Miler T Lee

**Affiliations:** Department of Biological Sciences, University of Pittsburgh, Pittsburgh, PA 15260, USA; Department of Biological Sciences, University of Pittsburgh, Pittsburgh, PA 15260, USA; Department of Biological Sciences, University of Pittsburgh, Pittsburgh, PA 15260, USA

## Abstract

RNA sequencing (RNA-seq) is extensively used to quantify gene expression transcriptome-wide. Although often paired with polyadenylate (poly(A)) selection to enrich for messenger RNA (mRNA), many applications require alternate approaches to counteract the high proportion of ribosomal RNA (rRNA) in total RNA. Recently, digestion using RNaseH and antisense DNA oligomers tiling target rRNAs has emerged as an alternative to commercial rRNA depletion kits. Here, we present a streamlined, more economical RNaseH-mediated rRNA depletion with substantially lower up-front costs, using shorter antisense oligos only sparsely tiled along the target RNA in a 5-min digestion reaction. We introduce a novel Web tool, Oligo-ASST, that simplifies oligo design to target regions with optimal thermodynamic properties, and additionally can generate compact, common oligo pools that simultaneously target divergent RNAs, e.g. across different species. We demonstrate the efficacy of these strategies by generating rRNA-depletion oligos for *Xenopus laevis* and for zebrafish, which expresses two distinct versions of rRNAs during embryogenesis. The resulting RNA-seq libraries reduce rRNA to <5% of aligned reads, on par with poly(A) selection, and also reveal expression of many non-adenylated RNA species. Oligo-ASST is freely available at https://mtleelab.pitt.edu/oligo to design antisense oligos for any taxon or to target any abundant RNA for depletion.

## Graphical Abstract

**Graphical Abstract:**
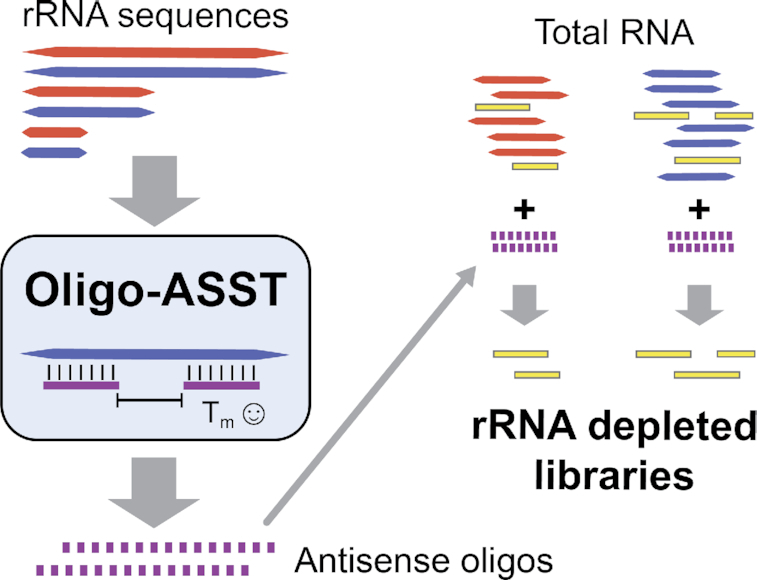
Oligo-ASST designs optimized antisense oligo sets to target RNAs for depletion.

## INTRODUCTION

High-throughput RNA sequencing (RNA-seq) has become a widespread method for measuring gene expression transcriptome-wide (1). Most RNA-seq studies focus on messenger RNA (mRNA); however, the vast majority of total RNA (>80%) (2,3) is ribosomal RNA (rRNA). Therefore, RNA-seq is commonly paired with methods to reduce the amount of rRNA included in sequencing libraries, to maximize the proportion of sequencing reads derived from genes of interest.

An effective, widely used strategy for enriching mRNA is polyadenylate (poly(A)) selection (poly(A)+) (1). In eukaryotes, most mRNAs encode 3′ poly(A) tails, which are used to select for and enrich mRNA pools using oligo(dT)-based methods (1,4). However, many applications cannot take advantage of this approach, notably transcriptomics in prokaryotes, whose mRNA largely lack poly(A) tails (3), but also many eukaryotic contexts as well. Methods that aim to quantify message fragments separated from poly(A) tails, such as RNA-seq on degraded RNAs (5,6), cap analysis gene expression (CAGE) (7) and ribosome profiling (8), require alternate rRNA depletion strategies. Some RNAs of interest, such as nascent pre-mRNA, some histone mRNA and many non-coding RNAs (ncRNAs), do not encode poly(A) tails (9), thus their expression levels are underrepresented in poly(A)+ RNA-seq libraries. Finally, since poly(A) tail length is variable, it is a challenge to distinguish changes in poly(A) status, e.g. due to the activity of deadenylases, from changes in RNA molecule number using poly(A)+ RNA-seq (10). Indeed, in animals such as *Xenopus* and zebrafish, the maternal mRNA contribution to the egg is largely deadenylated (11), thus poly(A) selection is not well suited to accurately measure the transcriptome in the early embryo (12–15).

An alternative to enrich for mRNAs in libraries is exon capture, in which samples are hybridized to oligo probes designed against the transcriptome of interest prior to sequencing (16). While particularly effective in clinical settings (17), commercial probe sets are costly and available for only select, well-annotated transcriptomes, limiting the feasibility of this method for many RNA-seq applications. Thus, methods to exclude unwanted RNA species have also been widely used, including electrophoretic size selection (18), digestion of highly abundant species in cDNA libraries using duplex-specific nucleases (19,20), and targeted rRNA depletion (21). In the latter approach, DNA oligos complementary to rRNA facilitate their removal prior to library construction. These oligos can be biotinylated for magnetic bead affinity purification (6,22,23), commercialized for some taxa in the Ribo-Minus and the now discontinued Ribo-Zero Gold kits.

Antisense oligos can be used in conjunction with RNaseH to digest DNA–rRNA hybrids (5,24). Several studies in both mammals and bacteria have shown that RNaseH-mediated rRNA depletion is efficient, resulting in sequencing libraries with minimal rRNA derived reads (5,23–27). Commercial solutions have emerged for select taxa; however, the ease of this method allows it in principle to be readily adapted to any taxon, with the primary challenge being the design and acquisition of the 50 nt oligos that tile the specific rRNA sequences encoded in its transcriptome. Although rRNA sequences are generally well conserved between species relative to other genes, nucleotide differences and variable regions at even modest evolutionary distances (28) pose a challenge for reusing oligos designed for one taxon to effectively perform rRNA depletion in another.

Here, we present an optimized strategy for RNaseH-mediated rRNA depletion suitable for RNA-seq library construction that reduces up-front oligo costs by as much as 81%. Using *Xenopus laevis* and zebrafish (*Danio rerio*) embryos as test cases, we demonstrate that depletion using short (39–40 nt) antisense DNA oligos sparsely tiled along rRNA, coupled with a 5-min digestion, effectively produces RNA-seq libraries with <5% rRNA-derived reads, on par with poly(A) selection. We show that divergent rRNAs can be simultaneously digested with partially overlapping oligo pools that target regions of high sequence similarity, facilitating the design of flexible, cross-taxon reagents for rRNA depletion. Finally, we introduce a web tool, Oligo-ASST, that simplifies oligo design, allowing this approach to be easily adapted to any taxon or to target any other abundant RNAs for depletion.

## MATERIALS AND METHODS

### Animal husbandry

All animal procedures were conducted under the supervision and approval of the Institutional Animal Care and Use Committee at the University of Pittsburgh. *X. laevis* adults (NASCO NXR_0.0031) were housed in a recirculating aquatic system (Aquaneering) at 18°C with a 12/12 h light/dark cycle. Frogs were fed twice weekly with Frog Brittle (NASCO #SA05960(LM)M). *Danio rerio* (zebrafish) were housed in a recirculating aquatic system (Aquaneering) at 27°C with a 14/10 hour light/dark cycle and fed freshly hatched *Artemia spp*. nauplii twice daily, supplemented with TetraMin Tropical Flakes and dried krill.

### Sample collection

To obtain *Xenopus laevis* embryos, sexually mature females were injected with 1000 IU human chorionic gonadotropin into their dorsal lymph sac and incubated overnight at 16°C. In the morning, females were moved to room temperature where they laid eggs within an hour of being moved. Sexually mature males were euthanized by 30 minute submersion in 3.6 g/l tricaine-S (MS-222), pH 7.4, and testes were dissected. Cleaned testes were stored up to a week in L-15 medium at 4°C. Eggs were collected and artificially inseminated in MR/3 (33 mM NaCl, 0.6 mM KCl, 0.67 mM CaCl_2_, 0.33 mM MgCl_2_, 1.67 mM HEPES, pH 7.8) (29). Zygotes were de-jellied (30) in MR/3 pH 8.5, with 0.3% β-mercaptoethanol with gentle manual agitation, neutralized with MR/3 pH 6.5, washed twice with MR/3 and incubated in MR/3 at 23°C until desired developmental stage.

Zebrafish embryos were obtained from natural mating of TUAB strain fish 6–12 months old. Mating pairs were selected randomly from a pool of 24 males and 24 females ≥1 month since last breeding. Zebrafish were isolated in mating pairs overnight at room temperature in divided tanks. Dividers were removed the following morning, and eggs were collected in egg water (60 μg/ml Ocean salt in RO water) and incubated at 28.5°C until the desired developmental stage.

To obtain fin clips, adult zebrafish were anesthetized in 500 mg/l MS-222 in system water for 2–5 min until gills stopped moving, then one lobe of the caudal fin was clipped. Fish were transferred to fresh system water for recovery.

### Total RNA extraction

For *X. laevis*, two embryos were pooled for RNA extraction; for *D. rerio*, 20 embryos or 10 fin clips were pooled. Samples were snap frozen in a 1.5 ml tube and homogenized with a pestle in 500 μl of TRIzol Reagent (Invitrogen #15596026) followed by 100 μl of chloroform. Tubes were centrifuged at 18 000 × g at 4°C for 15 min, the aqueous phase was transferred to a fresh tube with 340 μl of isopropanol and 1 μl of GlycoBlue (Invitrogen #AM9515), then precipitated at −80°C for 1 h. Precipitated RNA was washed with cold 75% ethanol and resuspended in 50 μl of nuclease-free water. Concentration was determined by NanoDrop. RNA was stored at −80°C until use.

### Antisense oligo design

The oligo tiling program is written in Python3. Each tiled oligo is defined by the start and end position of the complementary region in the target sequence (e.g. an rRNA). The algorithm assigns oligo positions left to right in a greedy fashion, such that each oligo is the maximum distance from the previous placed oligo while satisfying the parameter constraints – by default, melting temperature (*T*_m_) between 70 and 80°C, length between 39–40 nucleotides (nts), and maximum untiled region of 30 nts. If no oligo exists that satisfies these constraints, the oligo with closest *T*_m_ to the ideal range is retained. The maximum untiled region is iteratively adjusted to take into account the remaining sequence length. Melting temperature is calculated using the nearest-neighbor method (31,32) with RNA-DNA parameters (33): *T*_m_ (°C) = (Δ*H* − helix_initiation_energy)/(Δ*S* + *R*•ln(1/[oligo]) + 16.6 log_10_([Na^+^]) − 273.15. This assumes oligo concentration is in excess of template, and is set to a conservatively low 50 mM compared to the oligo concentrations we use (100–400 mM), which will tend to slightly underestimate *T*_m_ by <2°C. Na^+^ concentration is set to 200 mM, and helix initiation energy for RNA-DNA hybrids is estimated as −3.1 kcal/°K•mol (33). At the time of writing, Banerjee *et al.* published improved RNA-DNA parameters (34), which may yield slightly different predicted *T*_m_s compared to the old parameters used here; future software updates will incorporate these parameters. Once the entire target sequence is tiled, a second refinement phase adjusts each oligo position within the window defined by the upstream and downstream gaps, to yield maximized distances from upstream and downstream oligos within the ideal *T*_m_ range.

To find shared oligo pools between two or more unaligned target sequences, oligo tiling proceeds as above for the first sequence. For each subsequent sequence, oligos from the first set with exact complementary matches are selected, then the remaining untiled regions are subjected to the tiling procedure as above. To find shared oligo pools between aligned target sequences, a consensus sequence from the alignment is used for the first round of oligo tiling to generate the candidate common oligos for subsequent rounds of tiling for each individual sequence. If wildcards bases are allowed, the consensus sequence will incorporate IUPAC wildcard bases. Wildcard-containing oligos are retained if the number of possible target sequences does not exceed the threshold specified by the user (e.g., an oligo with two wildcard positions, R and Y, would target four different sequences encoding all combinations of C/T and A/G at the complementary positions, respectively). We ordered wildcard-containing oligos directly from the manufacturer as oligo mixtures, but they can also be ordered as individual unambiguous oligos and subsequently mixed.

Oligos for *X. laevis* rRNA were designed individually for 28S (X02995.1:3836–7917), 18S (X02995.1:1030–2854), 5.8S (X02995.1:3412–3573), 16S (M10217.1:3093–4723), and 12S (M10217.1:2205–3023). Aligned consensus oligos were designed for the maternal and somatic 5S (maternal: M10635:352–471, somatic: J01009.1:607–726) (35). The alternate mitochondrial rRNA sequences encoded in our samples are HM991335.1:1086–2726 for 16S and HM991335.1:69–1016 for 12S. *COX2* and *COX3* sequences were obtained from the X. laevis v9.2 genome assembly, chrM:9109–9796(+) and chrM:10711–11491(+) respectively. For zebrafish, aligned consensus oligos were designed for maternal and somatic 28S (chr4:77556054–77560323(−) and chr5:820029–824137(−) respectively), maternal and somatic 18S (chr4:77561203–77563141(−) and chr5:824921–826807(−) respectively), maternal and somatic 5.8S (chr4:77560653–77560810(−) and chr5:824488–824644(−) respectively), and maternal and somatic 5S (chr4:41890222–41890340(−) and chr18:30048558–30048676(−) respectively), according to previous annotations (36,37). Individual oligos sets were designed for 16S (chrM:1020–1971(+)) and 12S (chrM:2043–3725(+)). All coordinates are from the GRCz11 genome build.

Oligos were ordered from Thermo Fisher as individual dry, desalted tubes at 25 nmol scale. At the time of writing, value oligo pricing (≥25 oligos with length ≤40 nts) was US$4.64 per oligo, thus a full *X. laevis* set (137 oligos) would cost ∼US$636. In contrast, standard 50mer oligos are US$19 each (without institutional discount), thus the 176 oligos required for full tiling would total US$3344. With an institutional discount, this would likely still be >US$1400.

### RNaseH-mediated depletion

Individual dry oligos were resuspended to 1000 μM. For *X. laevis*, a 10X working stock for nuclear rRNA (28S, 18S, 5.8S, maternal and somatic 5S) was created by pooling 1 μl of each of the 96 oligos and diluting to 4 μM per individual oligo (250 μl total volume, 384 μM total oligo concentration). At 1× concentration in 10 μl, each oligo is at 400 nM, which we estimate to be 10-fold in excess of its target in 1 μg of total RNA: assuming 80% of total RNA is derived from 40S rRNA (28S, 18S, 5.8S in equimolar amounts) and 28S rRNA is 2× the length of 18S+5.8S, this corresponds to ∼530 ng of the ∼4000-nt 28S rRNA, or ∼41 nM in 10 μl. A similar stock of 41 oligos targeting the less abundant mitochondrial rRNA (16S, 12S) was prepared at 1 μM per individual oligo. For the parameter evaluation libraries, 15 of the original mitochondrial rRNA oligos, seven new mitochondrial rRNA oligos, seven *COX2* oligos and nine *COX3* oligos were pooled at 1 μM per oligo, and this stock was used with the original nuclear rRNA oligo stock for depletion. For zebrafish, separate working stocks for maternal nuclear (112 oligos at 4 μM per oligo), somatic nuclear (109 oligos at 4 μM per oligo), and mitochondrial rRNA (42 oligos at 1 μM per oligo) were similarly constructed. Maternal and somatic nuclear pools were then proportionally mixed according to developmental stage (1:0 for two-cell, 1:1 for 28hpf, 0:1 for adult) (37). Hybridization procedure was based on Adiconis *et al.* (24) with slight modifications: 1 μl of the nuclear pool (final concentration 0.4 μM per oligo) and 1 μl of the mitochondrial pool (final concentration 0.1 μM per oligo) were combined with 1 μg of total RNA (and optionally 150 ng of *in vitro* transcribed mCherry mRNA) in a 10 μl buffered reaction volume (100 mM Tris–HCl pH 7.4, 200 mM NaCl, 10 mM DTT), heated at 95°C for 2 min and cooled to 22°C at a rate of 0.1°C/s in a thermal cycler. Next, 10 U of thermostable RNaseH (NEB #M0523S) and 2 μl of provided 10× RNaseH buffer were added and volume brought to 20 μl with nuclease-free water. We achieved the best results with NEB thermostable RNaseH compared to other commercial RNaseH products. The reaction was incubated at either 45°C or 65°C for 5 or 30 min, then 5U of TURBO DNase (Invitrogen #AM2238) and 5 μl of provided 10× buffer was added, volume brought to 50 μl with nuclease-free water and incubated at 37°C for 30 min. Oligos were omitted from input control samples prior to heating and enzyme addition. For visualization, 12.5 μl of each reaction was run on a 1% formaldehyde 1.2% agarose gel in MOPS buffer (10× stock: 200 mM MOPS, 50 mM NaAc, 10 mM Na_2_EDTA, pH 7.0) at 80V. Gels were stained with SYBR Gold (Invitrogen #S11494) for 30 minutes. For qRT-PCR and RNA-seq, the reaction was purified and size selected to >200 nts using Zymo Clean and Concentrator-5 (Zymo #D4013) according to manufacturer's protocol, eluting in 10 μl of nuclease-free water. RNA was stored at −80°C.

### Poly(A) selection

Polyadenylated mRNA was selected using the NEBNext Poly(A) mRNA Magnetic Isolation Module (NEB #E7490L) according to the manufacturer's protocol: 1 μg of total RNA was denatured at 65°C for 5 min then hybridized to buffered dT magnetic beads at room temperature for 2 min. Selected RNA was eluted in 50 μl of Tris buffer at 80°C for 2 min and rehybridized to the same beads for a second round of selection at room temperature for 2 min. Re-selected RNA was eluted in a final volume of 17 μl of Tris buffer and stored at −80°C until further use.

### Quantitative reverse transcription PCR (qRT-PCR)

For first strand synthesis, a 20 μl reaction consisting of Zymo-cleaned RNA (∼50 ng), dNTP (1 μM), random primer (NEB #S1330S) (3 μM), and DTT (10 mM) was incubated at 65°C for 5 min then transferred to ice for 2 min. 1 μl of SuperScript III reverse transcriptase enzyme and 8 μl of 5× buffer (Invitrogen #18080085) were added to a final reaction volume of 40 μl, incubated at 42°C for 90 min, then heat inactivated at 70°C for 15 min. Initial samples for the *X. laevis* 28S qRT-PCR were column purified (Qiagen #28704) and used at full concentration for qRT-PCR; subsequent samples were used directly at 1:10 dilution for qRT-PCR based on the results of a 4-sample, 1:5 dilution calibration curve analysis. qRT-PCR was performed in triplicate using 10 μl reactions (2.5 μl of cDNA, 5 μM of each forward and reverse primers, and 2× SYGreen mix (Genesee #17-505B)). qPCR was performed on QuantStudio 3 (Applied Biosystems) with an initial heat activation at 50°C for 2 min and then 95°C for 10 min. The reactions were cycled at 95°C for 15 s and 60°C for 1 min for 40 cycles. Specificity was determined via a 3-stage melt curve analysis conducted at 95°C for 15 s, dropped to 60°C for 1 min, and then raising the temperature from 60°C to 95°C at 0.1°C/s. No-template negative controls were run for each primer pair. Data analysis was conducted in Design and Analysis Application v1.5.1 (Thermo Fisher) and C_t_ values were calculated automatically from that application. Each NTC sample resulted in a C_t_ > 34. Experimental samples resulted in C_t_ values ranging between 14 and 33. ΔC_t_ values were calculated from the average of three technical replicates for each sample using mCherry as the reference gene and plotted ΔΔC_t_ values represent depletion conditions ΔC_t_ over input RNA control ΔC_t_. Statistical comparisons were done using two-tailed paired *t* tests on ΔC_t_ values (each treated sample is paired with the input RNA that was used for treatment). Primers were: **28S** (F-TGTGATTTCTGCCCAGTGCT; R-GACGAGGCATTTGGCTACCT, amplicon: 107 bp), **16S** (F-TCCAAAAACCTAGCATTCCAATTAT; R-TTTCATCTTTCCTTACGGTACTTTTTC, amplicon: 140 bp), **mCherry** (F-GCCCCGTAATGCAGAAGAAG; R-TCAGCTTCAGCCTCTGCTTG, amplicon: 105 bp), **sub1.L – XM_018266533.1** (F-AGCAGGAGAAATGAAGCCAGG-exon 4; R-CCGACATCTGCTCCTTCAGT-exon 5, amplicon: 80 bp) (38); **helb.L – XM_018252426.1** (F-TTTCCAGGGTTCAGAAGAGGAG-exon12/13 junction; R-TGCTATGGCTTCACCCAACT-exon 13, amplicon: 148 bp); **nudt15.L – XM_018245539.1** (F-CCTGAGAAAAACGAAGGTTGGAA-exon3/4 junction; R-TGGATTGTAGCCTTGCTGCT-exon 4, amplicon: 105 bp). Primer specificity was verified using NCBI Primer-BLAST.

### RNA sequencing

Strand-specific RNA-seq libraries were constructed using the NEB Ultra II RNA-seq library kit (NEB #E7765) according to manufacturer's protocol with fragmentation in first-strand buffer at 94°C for 15 min. Following first and second strand synthesis, DNA was purified with 1.8× AmpureXP beads (Beckman #A63880), end repaired, then ligated to sequencing adaptors diluted 1:5. Ligated DNA was purified with 0.9× AmpureXP beads and PCR amplified for 8 cycles, then purified again with 0.9× AmpureXP beads. Libraries were verified by Qubit dsDNA high sensitivity (Invitrogen #Q32851) and Fragment Analyzer prior to multiplexed paired-end sequencing on an Illumina NextSeq 500 at the Health Sciences Sequencing Core at Children's Hospital of Pittsburgh.

### RNA-seq data analysis

RNA-seq reads were mapped to the X. laevis v9.2 or GRCz11 (zebrafish) genomes using HISAT2 v2.0.5 (39) (–no-mixed –no-discordant) and assigned to genes (Xenbase v9.2 models for *X. laevis* and Ensembl r99 for zebrafish) using featureCounts v1.5.1 (40) in reversely-stranded paired-end mode with default parameters. To more accurately quantify rRNA levels in the *X. laevis* genome, due to poor assembly at the 40S rDNA locus, we additionally aligned to a separate HISAT2 index consisting of only the 40S (X02995.1) and 5S (J01009.1) sequences. Coverage plots were generated using BEDTools v2.25.0 genomeCoverageBed (41) and visualized on the UCSC Genome Browser (42). To annotate histone mRNA, *X. laevis* and zebrafish protein sequences were curated from HistoneDB 2.0 (43) and used to construct NCBI BLAST blastx databases (44). Xenbase and Ensembl zebrafish mRNA hits with *E*-value < 1e–40 were annotated as histones. To correlate oligo features with depletion efficiency, oligo positions or gap positions were converted to a genomic coordinate BED file and used to calculate RNA-seq coverage per feature using the BEDTools multicov command. To estimate oligo off targeting, we constructed an NCBI BLAST database of oligos and performed a gapless blastn (word size 5, reverse strand only, *E*-value threshold 100) querying every transcript in the transcriptome, retaining the best oligo hit per transcript. Bit score and number of identities were each plotted against log2 TPM fold difference in rRNA depleted versus poly(A)+ samples, for all genes with TPM ≥ 1 in either sample. All plots and analyses were generated using R-3.4.4.

## RESULTS

### Sparse antisense oligo tiling effectively depletes rRNA

**Figure 1.**
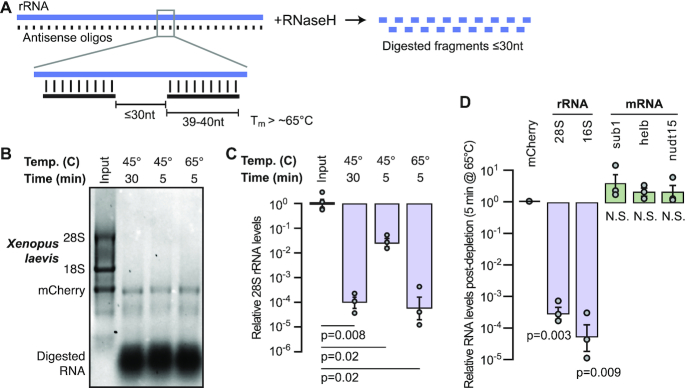
(**A**) Schematic of rRNA depletion strategy using 39–40 nt antisense oligos spaced ≤ 30-nt apart. (**B**) *X. laevis* stage 0 total RNA (input, lane 1) and with rRNA depletion using different reaction conditions visualized on a 1% formaldehyde 1.2% agarose gel. In vitro transcribed mCherry mRNA was spiked into the input RNA prior to digestion. (**C**) qRT-PCR comparing 28S rRNA levels in *X. laevis* stage 0 total RNA (input, left) versus depletion conditions normalized to mCherry. *P* values are from two-tailed paired *t* tests comparing depleted samples to their corresponding total RNA input. (**D**) qRT-PCR measuring mCherry-normalized rRNA and mRNA levels in *X. laevis* stage 0 rRNA-depleted samples divided by levels in untreated samples. P values are from two-tailed paired t tests for each gene comparing depleted samples to their corresponding total RNA input. N.S. = not significant.

Previous RNaseH-based depletion methods used 50-nt DNA antisense oligomers that completely tile target RNA species (5,24,25) (Supplementary Figure S1a). We reasoned that for many applications, e.g. RNA-seq for non-degraded samples, tiling with gaps should be effective if the resulting fragments are short enough to be filtered out by size selection prior to cDNA generation. To test this strategy, we designed 39–40 nt oligos spaced ≤30 nt apart (Figure 1A) to tile the *X. laevis* nuclear (28S, 18S, 5.8S, 5S) and mitochondrial (16S, 12S) rRNA. The gaps would yield fragments ≤30 nts if the oligos induce digestion to completion, or ∼70 nts with partial digestion if the flanking oligos each induce cleavage in the center (Supplementary Figure S1b), comparable to the size of tRNAs. By including gaps, we were able to select oligos with high predicted melting temperatures (*T*_m_), to ensure that they are hybridized with their targets at the digestion reaction temperature. With the aid of a computational tool we created (see below), we designed most oligos with *T*_m_ between 65 and 87°C, with the exception of seven oligos targeting 16S rRNA with *T*_m_ between 58 and 64°C due to sequence constraints. By comparison, an end-to-end 50-nt tiling strategy would produce oligos with *T*_m_ between 52 and 94°C. Shortening the oligo lengths allowed us to take advantage of value oligo pricing, and the overall strategy used 137 individual oligos tiling 5434 total bases (Supplementary Table S1), compared to 176 oligos and 8639 bases (a 37% reduction) for the end-to-end 50-nt strategy. This led to an 81% reduction in total oligo cost according to list prices (Materials and Methods).

We combined aliquots of all of the nuclear rRNA-targeting oligos into a working stock of 4 μM for each of the individual oligos and a similar stock for mitochondrial rRNA-targeting oligos at 1 μM. At 1X, the oligo pools target ∼1 μg of total RNA, such that each oligo is in ∼10-fold excess of its rRNA target (Methods). To test the efficacy of the oligo pools, we subjected Nieuwkoop and Faber (NF) embryonic stage 0 *X. laevis* total RNA to RNaseH treatment and visualized the digested RNA, without any cleanup, on a 1% formaldehyde-agarose gel. We tested previously published reaction parameters (45°C for 30 min) using thermostable RNaseH along with two other conditions that reduced reaction time (45°C for 5 min) and additionally increased reaction temperature (65°C for 5 min) (Figure 1B). To test specificity of the treatment for rRNA, we spiked 150 ng of *in vitro* transcribed mCherry mRNA into each reaction. All three reaction conditions were effective, eliminating the upper bands corresponding to the 28S (4082 nts) and 18S rRNA (1825 nts) while leaving the mCherry (1037 nts) band intact (Figure 1B). A diffuse band migrating at ∼500 nts is also intact in the digested samples, which likely corresponds to highly abundant histone mRNA species, based on inspection of RNA-seq datasets. A large mass that is likely digested RNA and DNA oligos is visible at the bottom of each lane at <50 nts (Figure 1B), which we expect to be largely excluded if size selection is performed after digestion.

To precisely quantify the rRNA depletion, we subjected samples in triplicate to quantitative reverse transcription PCR (qRT-PCR) probing for 28S rRNA. All three depletion conditions significantly reduce the level of 28S rRNA compared to untreated RNA, with the 45°C/30 min and 65°C/5 min reactions reducing 28S rRNA levels by 99.99% (*P* < 0.05, two-tailed paired *t* test) (Figure 1C, Supplementary Table S2)—the optimal reaction temperature for the thermostable RNaseH is 65°C, and these results demonstrate that digestion is rapid at this temperature.

To assess the effects of rRNA depletion on mRNA as compared to rRNA, we performed qRT-PCR on treated (RNaseH 65°C/5 minutes) versus untreated total RNA, probing for embryonic mRNA expressed at low to moderate levels based on previous RNA-seq studies (45)—*sub1.L* (133 transcripts per million (TPM)), *helb.L* (5 TPM), and *nudt15.L* (1 TPM)—along with 28S and mitochondrial-encoded 16S rRNA, normalizing to mCherry spike in. Both rRNA species were significantly depleted in treated versus untreated samples (*P* < 0.001, two-tailed paired *t* test) (Figure 1D, Supplementary Table S3), while the mRNA levels were not significantly different (*P* > 0.1, two-tailed paired *t* test) (Figure 1D). Taken together, we find that the optimized oligo design effectively and specifically degrades targeted rRNA, using streamlined reaction times.

### Optimized rRNA depletion yields high quality RNA-seq libraries

**Figure 2.**
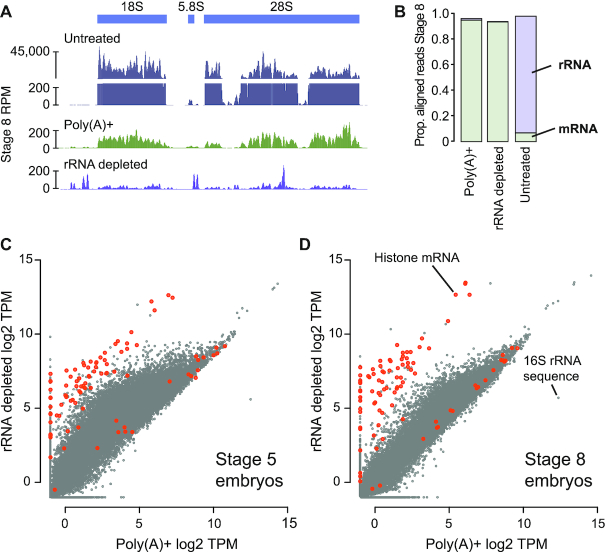
(**A**) Genome browser tracks comparing read coverage at the *X. laevis* 40S rDNA locus in untreated total RNA, poly(A)+ and rRNA depleted RNA-seq libraries from stage 8 embryos. Y-axis is discontinuous for the total RNA sample. (**B**) Stacked barplots showing proportion of aligned reads deriving from mRNA or lncRNA (green) versus rRNA (purple) in untreated, poly(A)+ and rRNA depleted RNA-seq libraries. (**C**, **D**) Biplots comparing log_2_ TPM expression levels and poly(A)+ and rRNA-depleted libraries at stage 5 and 8, respectively. Histone genes are highlighted in orange. RPM = reads per million, TPM = transcripts per million.

Next, we sought to determine whether our depletion strategy could be used to construct high quality RNA-seq libraries. We collected total RNA from two different *X. laevis* embryonic stages (NF 5 and 8) and performed either rRNA depletion (65°C/5 min) or poly(A)+ selection. We column size-selected the rRNA depleted samples to enrich for RNAs >200 nts, thus excluding digested rRNA fragments, then built Illumina strand-specific libraries and sequenced each sample to 5–10 million read pairs. Both the poly(A)+ and rRNA depleted samples show a >100-fold reduction in reads aligning the 40S rDNA locus compared to unselected total RNA (Figure 2A). Indeed, overall <0.3% of reads derive from rRNA while >90% of reads align to annotated mRNA or long non-coding RNA (lncRNA) in both the poly(A)+ and rRNA depleted samples, compared to <7% mRNA reads for unselected total RNA (Figure 2B, Supplementary Figure S2a). This efficiency is comparable to previous rRNA depletion strategies using full 50-nt end-to-end oligo tiling (5,24,25), demonstrating that gapped oligo tiling can achieve highly efficient rRNA depletion.

Transcriptome wide, expression levels correlate well between poly(A)+ and rRNA depletion for most genes (Figure 2C, D, Supplementary Table S4). However, at stage 5, a population of transcripts shows elevated apparent levels with rRNA depletion compared to poly(A)+ (Figure 2C). Indeed, the maternal RNA contribution to the egg is largely deadenylated, with poly(A) tails lengthening during early embryonic stages through cytoplasmic polyadenylation (11). Thus, rRNA depletion avoids the depressed expression levels arising from inefficient capture of mRNA with short poly(A) tails, typical of poly(A)+ RNA-seq (12–15). By the mid-blastula transition (NF stage 8), poly(A) tails are longer, so poly(A)+ and rRNA depletion yield comparable expression values for these mRNA (Figure 2D). However, some RNA species are still better represented in the rRNA depletion libraries, suggesting these transcripts lack poly(A) tails. Indeed, replication-dependent histone mRNA encode 3′ stem loops instead of poly(A) tails (46), and we find these transcripts are much more efficiently sequenced with rRNA depletion (Figure 2C and D). Thus, our optimized rRNA depletion strategy effectively quantifies expression levels of both the adenylated and non-adenylated transcriptome.

To determine if the antisense oligos were inducing off-target depletion of mRNA, we performed BLAST alignment for each oligo against the annotated transcriptome and found that the majority of transcripts have low sequence similarity to any rRNA-targeting oligo: 99.4% of transcripts have <50% sequence identity to any oligo. However, we did identify three predicted transcripts that have high similarity to rRNA sequences, which are likely falsely annotated mRNA (Supplementary Figure S2b). We additionally found no correlation between sequence similarity to an oligo and RNA-seq fold difference between the rRNA-depleted and poly(A)+ samples, whereas if significant off-target digestion were occurring, we would expect higher BLAST similarity to be correlated with a lower sequenced expression level with rRNA depletion compared to poly(A)+ (Supplementary Figure S2c and d). Thus, antisense-oligo depletion using 39–40mers can achieve high efficiency and on-target specificity.

### Oligo spacing and melting temperature affect depletion efficiency

To further evaluate antisense oligo design parameters, we sequenced additional libraries using variations to our original depletion strategy. First, we observed that our *X. laevis* seemed to be encoding a mitochondrial variant that differs from the xenLae 9.2 reference genome. However, the alternate sequence spanning the 12S and 16S rRNA genes was included in the genome sequence as a scaffold (chrUn_NW_016695825v1). Although the 12S and 16S sequences are each >97% identical between the variants, the alternate 12S gene has a divergent 5′ portion that was not targeted by our original oligo pool (Supplementary Figure S2e). By adding two new oligos to the depletion reaction, we effectively depleted the alternate 12S rRNA sequence in a new library (Supplementary Figure S2f).

**Figure 3.**
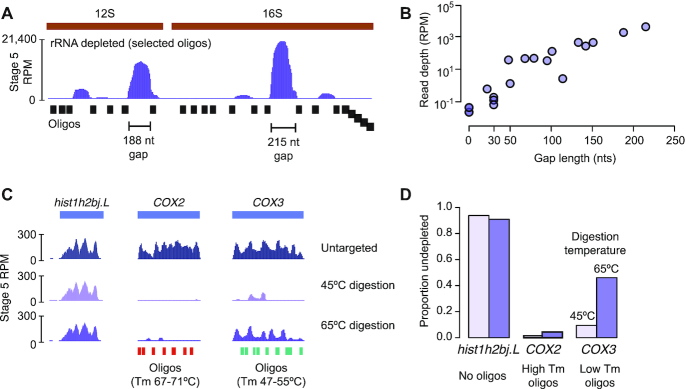
(**A**) Genome browser track showing read coverage over *X. laevis* 12S and 16S rDNA, illustrating the effect of rRNA depletion using variably spaced oligos (bottom). The two largest untiled regions are highlighted. (**B**) Biplot showing sequencing read depth at the center of each untiled region (gap) over 12S and 16S as a function of length of the region. (**C**) Genome browser tracks showing read coverage for hist1h2bj.L, not targeted for depletion; COX2, targeted using oligos with high melting temperature (*T*_m_); and COX3, targeted using oligos with low *T*_m_. Depletion reactions at 45°C for 30 min and 65°C for 5 min are compared to a reaction lacking the gene-targeting oligos. (**D**) Barplot for the three genes as in (C) showing the ratio of RNA-seq transcripts per million in depletion reactions (45°C left bars, 65°C right bars) over the non depleted condition. RPM = reads per million.

To measure the effect of oligo spacing on depletion efficiency, we selectively omitted oligos from the 12S and 16S rRNA targeting pool to leave gaps of up to 215 nts untiled by oligos. We additionally tiled the 3′ section of 16S rRNA end-to-end with 50mer oligos to resemble a traditional design strategy (Supplementary Tables S5 and S6). We found that increasing the untiled gap size is correlated with poorer depletion efficiency (i.e., higher read depth), demonstrating that larger digested RNA fragments are less efficiently excluded during column clean up (Figure 3A and B). Conversely, there was a negligible difference in efficiency when gaps were eliminated, suggesting that a tiling strategy with moderate gaps (≤30 nts) is comparable to end-to-end tiling for rRNA-depleted sequencing libraries when paired with size selection (Figure 3A and B).

Finally, in our original depletion we used oligos with melting temperatures near or above the 65°C reaction temperature, and we observed no correlation between oligo *T*_m_ and depletion efficiency (Supplementary Figure S2g). To further explore the relationship between oligo *T*_m_ and reaction temperature on depletion, we targeted two highly abundant mRNA, *COX2* and *COX3* with high-*T*_m_ (67.2°C ≤ *T*_m_ ≤ 71°C) and low-*T*_m_ (47.4°C ≤ *T*_m_ ≤ 55.2°C) oligos respectively (Supplementary Table S5). Performing digestion at 65°C for 5 min yielded strong depletion of *COX2* but less efficient depletion of *COX3*, suggesting that the low-*T*_m_ oligos are poorly hybridized to their targets at 65°C (Figure 3C and D). However, lowering the reaction temperature (45°C for 30 min) improved *COX3* depletion (Figure 3C and D) with no other effects on the rest of the transcriptome (Supplementary Figure S2h, Table S4). Thus, better depletion is achieved using oligos with melting temperatures at or above the digestion reaction temperature.

### Compact oligo pools can simultaneously target divergent rRNAs

Given the gapped design strategy, it is likely that some sequence differences in target RNAs would be tolerated, allowing oligo pools designed for the rRNAs of one taxon to be used for another closely related taxon. At greater sequence dissimilarity, we reasoned that shared oligos could be designed to target common subsequences between two or more RNAs, with gaps positioned over variable regions, avoiding the need to design completely separate reagents for rRNA depletion.

**Figure 4.**
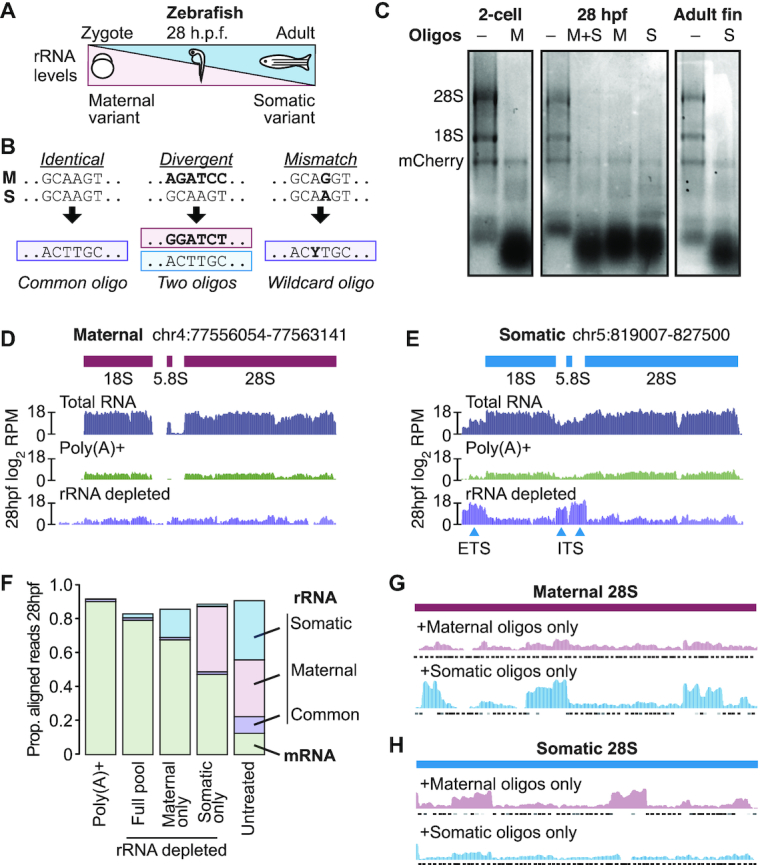
(**A**) Diagram illustrating the relative expression of the maternal and somatic nuclear rRNA variants over development. h.p.f. = hours post fertilization. (**B**) Schematic showing how oligos (bottom) can target similar sequences (top) between two RNAs. (**C**) Gels showing total RNA samples after rRNA depletion across three zebrafish developmental timepoints using only the maternal pool (M), only the somatic pool (S), or a mixture of the two pools (M+S), as compared to untreated input (−). In vitro transcribed mCherry mRNA was spiked into the input RNA prior to digestion. (D, E) Genome browser tracks comparing read coverage at the maternal (**D**) and somatic (**E**) 45S rDNA loci in untreated, poly(A)+ and rRNA depleted libraries from zebrafish 28 h.p.f. (**F**) Stacked barplots showing proportion of aligned reads deriving from mRNA (green) versus rRNA (blue, uniquely somatic; pink, uniquely maternal; purple, common) in untreated, poly(A)+ and rRNA depleted RNA-seq libraries. (**G**, **H**) Genome browser tracks comparing read coverage at the maternal (**G**) and somatic (**H**) 28S rDNA loci in rRNA depletion libraries depleted using only maternal (top row) or only somatic (bottom row) oligo pools from zebrafish 28 h.p.f. Targeted regions by each oligo pool are shown beneath each track.

To test this, we designed a combined oligo pool to target the two versions of the zebrafish nuclear rRNAs, which are 86% similar. Zebrafish encode maternal-specific 28S, 18S, 5.8S and 5S rRNAs that are deposited into eggs during oogenesis (36,37). After zygotic genome activation, distinct somatic rRNAs begin to be transcribed and slowly replace the maternal versions as the embryo develops (Figure 4A). Thus, to effectively deplete rRNAs in zebrafish embryos, both versions would need to be targeted. We aligned each rRNA sequence pair and designed 46 oligos that target identical regions between the maternal and somatic versions. To target regions that differed at only one position, we additionally designed 22 oligos containing a wildcard base (e.g. R to represent either A or G), which we ordered as mixtures of two oligos (Figure 4B). Finally, 44 and 41 additional oligos were required to target divergent maternal and somatic regions, respectively. In all, the combined design required 153 total oligos to together target both sets of nuclear rRNAs (Supplementary Table S7), compared to 201 total oligos for two independent sets.

We combined the common and unique oligos to create separate maternal and somatic pools each at 4 μM per individual oligo. We also created a mitochondrial rRNA-targeting pool at 1 μM per oligo (there is only one known version of the 16S and 12S rRNAs). In the 2-cell stage embryo, the maternal+mitochondrial pools effectively and specifically induce rRNA depletion from total RNA, which is entirely maternally derived (Figure 4C, left), while in adult fins, the somatic+mitochondrial pools are effective (Figure 4C, right). We additionally tested depletion in 28 h post fertilization (h.p.f.) embryos, which express roughly equal amounts of maternal and somatic rRNA (37). Neither the maternal pool nor the somatic pool alone was as effective as a 1:1 mixture of both pools: using only the maternal or somatic pools produced several RNA species between 300 and 800 nts, suggesting incomplete digestion (Figure 4C, middle).

To quantify this difference in efficiency, we constructed RNA-seq libraries at 28 h.p.f. rRNA depletion with the combined oligo pool effectively reduced the number of sequencing reads mapping to either the maternal or somatic rRNA loci compared to untreated total RNA, comparable to poly(A)+ (Figure 4D, E, Supplementary Figure S3a). We did observe elevated levels of reads mapping to the external and internal transcribed spacers of the full somatic 45S transcript (5′ ETS and two ITS regions; Figure 4E), which were omitted from the oligo design; as well as a small region of 16S rRNA where targeting was less efficient (Supplementary Figure S3b–d). Nonetheless, 79% of reads mapped to mRNA or lncRNA, compared to 90% for poly(A)+ (Figure 4F), and expression quantification was highly correlated between the two methods (Supplementary Figure S3e, Table S8). rRNA depletion additionally recovered highly expressed non-coding RNAs such as the signal recognition particle and 7SK RNAs, which are not efficiently sequenced with poly(A)+ (Supplementary Figure S3e).

In contrast, rRNA depletion using only the maternal or somatic pools was less efficient. By targeting only the maternal rRNA, 15% of the library is still rRNA, mapping to the somatic 45S locus; and by targeting only the somatic rRNA, 38% of reads derive from rRNA, corresponding to the maternal 45S locus. This leaves only 68% and 47% of the library mapping to mRNA+lncRNA, respectively (Figure 4F). Read coverage over the maternal 28S rRNA gene indeed shows a failure to digest sequence regions where the somatic oligos lack complementarity (Figure 4G), while a similar pattern is observed over the somatic 28S gene when only maternal oligos are used (Figure 4H). These results show that a full maternal+somatic targeting strategy is required to achieve a maximally effective rRNA depletion and demonstrate that a shared, compact oligo pool can efficiently target these divergent sequences simultaneously.

### The Oligo-ASST Web tool streamlines antisense oligo design

**Figure 5.**
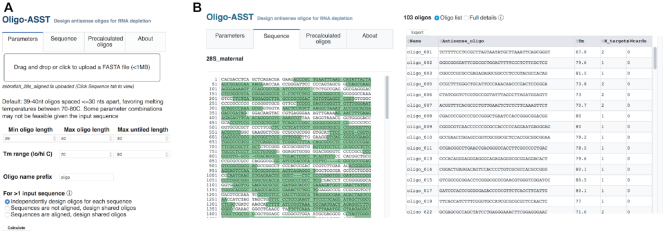
(**A**) The Oligo-ASST Web interface allows users to upload a FASTA file for target sequences and select parameters for oligo design. (**B**) Designed oligos are highlighted in a sequence viewer (left) and listed in the right pane in abbreviated form or with full details (not shown), which can be downloaded in text format.

The lack of an appropriate computational method to implement a gapped tiling strategy prompted us to build a Web tool called Oligo-ASST (which stands for Antisense Spaced Tiling) using the Python Dash v1.0 framework, available at https://mtleelab.pitt.edu/oligo. Oligo-ASST iteratively positions antisense oligos along a target sequence to maximize distance between consecutive oligos up to a threshold (e.g. 30 nts) while attempting to maintain a predicted *T*_m_ as close to the user defined target (e.g. 70°–80°C) as possible according to RNA/DNA duplex thermodynamic parameters (33). To design oligos, the user uploads one or more sequences in FASTA format (Figure 5A), selects oligo and gap length parameters according to their needs, then the resulting oligo sequences, coordinates and properties are displayed in the Web interface, where they can be downloaded in text format (Figure 5B, right, Supplementary Figure S4). Tiled positions are also highlighted on a Dash Bio Sequence Viewer (Figure 5B, left). Pre-calculated oligo sets, including the ones presented here, are also provided for download.

When multiple sequences are input, users can choose to design independent oligos per sequence or a shared set with common oligos targeting either identical subsequences or subsequences with one or more mismatches using wildcard bases. Sequences can be aligned beforehand using a tool such as MUSCLE (47) to improve identification of identical subsequences and yield a maximally compact oligo pool to target heterogeneous RNA species.

## DISCUSSION

Here, we demonstrate that a streamlined RNaseH digestion protocol using easily obtained reagents efficiently and cost-effectively achieves ribosomal RNA depletion (Supplementary File), which we estimate to be ∼US$8 per reaction. Our Web tool Oligo-ASST improves oligo design to use shorter antisense DNA oligos (39–40 nts) that tile rRNA target sequences with gaps, thereby reducing reagent cost compared to previous methods (5,24) while still producing high-quality RNA-seq libraries comparable to those constructed with poly(A) selection (Figures 2–4). Although there may be use cases where magnetic bead-based methods would be more appropriate, e.g. highly degraded RNA (6) or libraries where precise ends are required such as for ribosome profiling (48), for many RNA-seq applications RNaseH digestion should yield excellent results. In addition, since Oligo-ASST can also design compact oligo sets for treating multiple different rRNAs by targeting shared sequences, this protocol is especially advantageous for researchers to achieve rRNA depletion in diverse taxa.

We found that gaps of ≤30 nts untargeted by oligos did not seem to affect overall performance of rRNA depletion, suggesting that the majority of the resulting digested fragments are too short to be retained after column cleanup and size selection for the RNA-seq libraries. Thus, we have demonstrated that the previously standard full tiling strategy is unnecessary for a typical RNA-seq use case. Increasing allowable gap lengths leaves larger digested fragments and reduces the efficiency of the depletion (Figure 3A and B), though there may be applications for which this may be acceptable.

Our digestion reaction proceeds for only 5 min at 65°C using thermostable RNaseH, enabled by our use of oligos with *T*_m_s near or above 65°C. Reactions at lower temperatures produce comparable results, which would be beneficial when using oligos with lower *T*_m_s, e.g. to target AT-rich RNAs. Higher reaction temperatures could reduce the likelihood of off-targeting by the oligos, though we found no evidence that non-rRNA gene quantification was affected due to treatment in the transcriptomes we profiled (Figure 2C, D, Supplementary Figure S2b–d, h). Future optimizations to oligo design could avoid targeting regions with high sequence similarity to non-rRNAs, which is possible due to the flexibility of the gapped oligo tiling strategy.

For many taxa, designing new oligo sets should be straightforward with Oligo-ASST, given the availability of rRNA sequences in databases such as GenBank. For *Xenopus* and zebrafish, we found that the majority (75–86%) of rRNA reads in total RNA derive from 28S and 18S rRNA (Supplementary Figures S2a and S3a), thus targeting these alone would still yield RNA-seq libraries with a majority of non rRNA-reads. However, we did find a substantial fraction of reads mapping to 5.8S rRNA as well as the transcribed spacer regions ITS1, ITS2, and the 5′ ETS of the pre-rRNA; and the 16S and 12S mitochondrial rRNA, indicating that a maximally comprehensive oligo pool would target each of these eight sequences. Depending on the transcriptome of interest, it may also be valuable to target other abundant RNAs for depletion, e.g. the 7SK small nuclear RNA (Supplementary Figure S3e); inspection of existing RNA-seq libraries would reveal such RNA species. Indeed, Oligo-ASST is agnostic to RNA identity and can be used to design oligos that target arbitrary sequences (Figure 3C and D).

It is likely that targeting reagents will be somewhat robust to polymorphisms in the rRNA sequences, which may be especially prevalent in non in-bred strains and species. Indeed, we found that the mitochondrial rRNA variants encoded in our *X. laevis* were still effectively targeted by oligos designed against the reference mitochondrial genome (Figure 2, Supplementary Figure S2e, f), suggesting that a small number of mismatches are tolerated. However, Oligo-ASST can facilitate the design of oligos that map to non-variable regions, which as we show in zebrafish, allows a partially overlapping oligo pool to simultaneously target the two divergent sets of rRNAs in the zebrafish genome (Figure 4).

In conclusion, we have developed and optimized antisense oligo-based rRNA depletion for *X. laevis* and zebrafish RNA-seq libraries and provide a tool Oligo-ASST to design similar reagents for any other species. We anticipate this will be of benefit to researchers who need alternatives to poly(A) selection for RNA-seq, particularly those working with taxa that were inadequately served by previous rRNA depletion methods.

## DATA AVAILABILITY

The Oligo-ASST Web tool is available at https://mtleelab.pitt.edu/oligo.

Source code for the Web application and a command-line version of the program are available at https://github.com/MTLeeLab/oligo-asst.

All raw sequencing reads are deposited in the NCBI Gene Expression Omnibus under accession number GSE152902.

## Supplementary Material

gkaa1072_Supplemental_Files
