# Correction to ‘Optimized design of antisense oligomers for targeted rRNA depletion’

**DOI:** 10.1093/nar/gkab919

**Published:** 2021-09-30

**Authors:** Wesley A Phelps, Anne E Carlson, Miler T Lee

**Affiliations:** Department of Biological Sciences, University of Pittsburgh, Pittsburgh, PA 15260, USA; Department of Biological Sciences, University of Pittsburgh, Pittsburgh, PA 15260, USA; Department of Biological Sciences, University of Pittsburgh, Pittsburgh, PA 15260, USA

In the penultimate sentence in Materials and Methods - RNaseH-mediated depletion, the article listed an incorrect product number for the kit used to purify and size select rRNA-depleted samples. The correct product number is R1013 (Zymo RNA Clean and Concentrator-5).

The article (1) has been updated. The correction does not affect the results, discussion and conclusions presented in the article.

## References

[1] Phelps Wesley A., Carlson Anne E., Lee Miler T. Optimized design of antisense oligomers for targeted rRNA depletion. Nucleic Acids Research. 2021; 49:e5. 33221877 10.1093/nar/gkaa1072 PMC7797071

