# Supplementary material for: Optimized design of antisense oligomers for targeted rRNA depletion
Source: Nucleic Acids Res. 2020 Nov 22;49(1):e5. doi: 10.1093/nar/gkaa1072 (PMC7797071; doi:10.1093/nar/gkaa1072)
Supplement: gkaa1072_Supplemental_Files [file gkaa1072_supplemental_files.zip › Phelps20 - Supplementary Material.pdf]

Optimized design of antisense oligomers for targeted rRNA depletion

Wesley A. Phelps, Anne E. Carlson, Miler T. Lee

Supplementary Figures 1-4
Supplementary File: Protocol for RNaseH-mediated RNA depletion

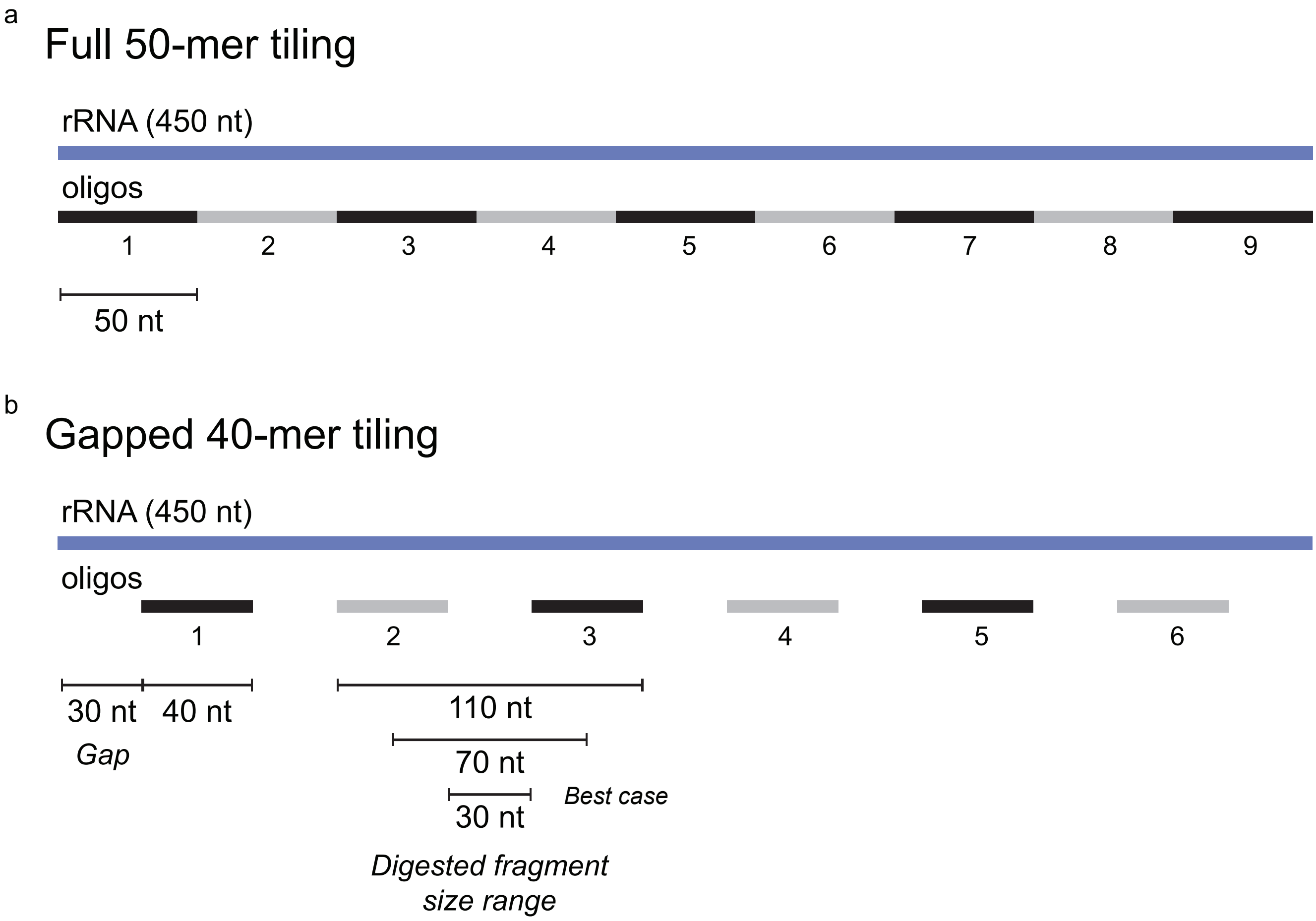

Supplementary Figure S1: a) Traditional design for rRNA depletion using 50mer antisense oligos that fully tile the target rRNA. b) A gapped tiling design using 40mer antisense oligos with 30 nt gaps uses fewer oligos to digest the target rRNA. The undigested fragments, optimally 30 nt in length, can be subsequently depleted from the sample using column-based size selection.

Supplementary Figure S1

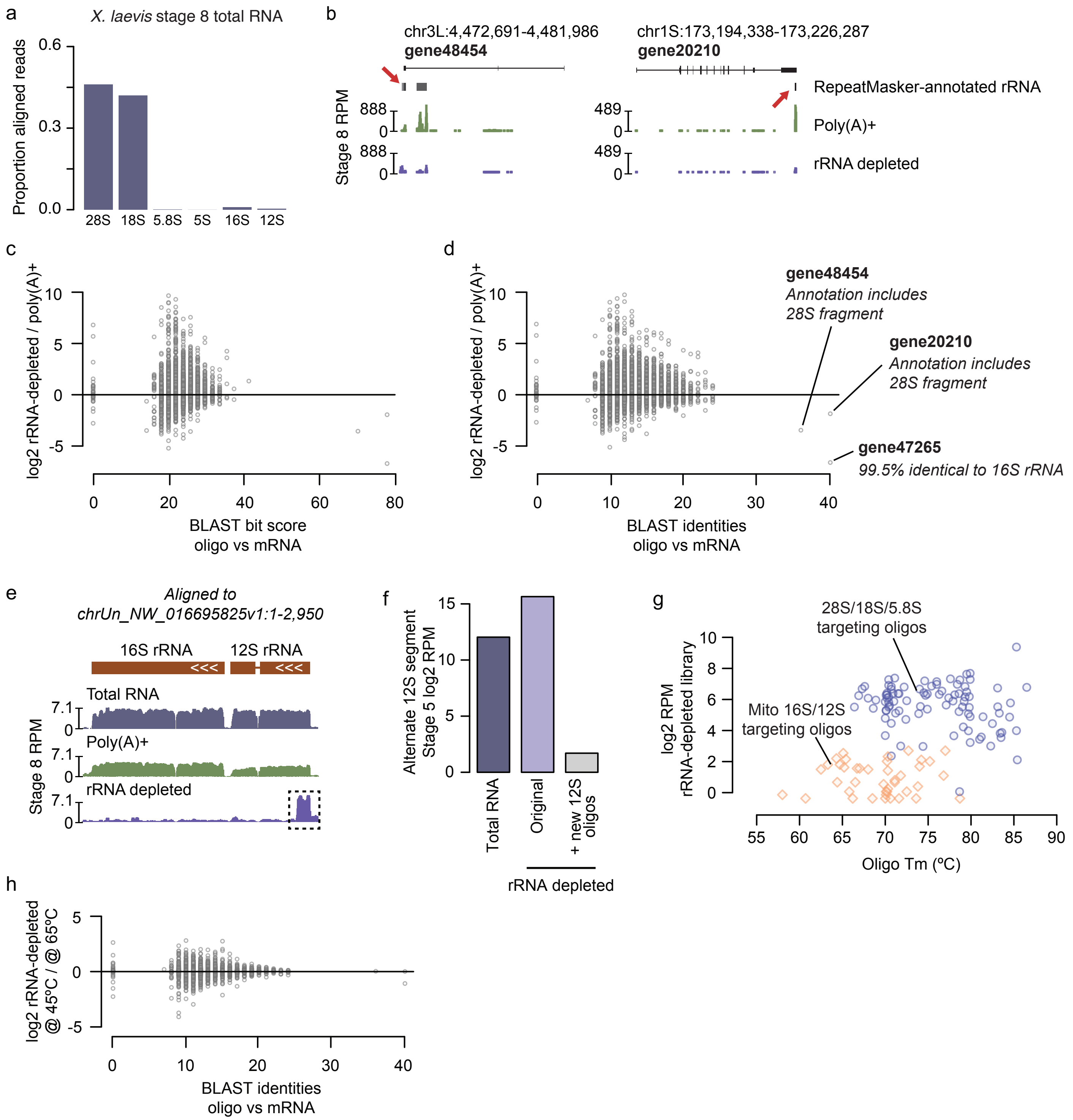

Supplementary Figure S2

Supplementary Figure S2: a) Barplots showing proportion of aligned reads from untreated total RNA mapping to rRNA species in *X. laevis*. b) Genome browser tracks illustrating rRNA sequences as annotated by RepeatMasker (red arrows) falling within annotated exons of two genes. c,d) Biplots showing the BLAST bit score (c) and number of identities (d) of each *X. laevis* mRNA to the most similar oligo sequence in the depletion pool, versus the log2 fold expression difference between the stage 8 rRNA-depleted and poly(A)+ samples. Three mRNA have very high similarity to oligos, which all contain sequences identical to rRNA, suggesting spurious annotations. e) Genome browser track of an unassembled *X. laevis* scaffold that harbors the sequences of alternate mitochondrial rDNA locus. The alternate 12S gene encodes a divergent 5´ end compared to the reference 12S gene, which fails to be depleted by the original oligo pool. f) Augmenting the original oligo pool with two oligos that target the alternate 12S 5´ divergent end effectively depletes this sequence. g) Biplot comparing oligo melting temperature (Tm) to log2 RPM coverage of the region targeted by each oligo in the *X. laevis* stage 8 rRNA depleted sample. Blue circles are nuclear rRNA-targeting oligos, orange diamonds are mitochondrial rRNA-targeting oligos. h) Same as (d) except comparing depletion at 45ºC to depletion at 65ºC. RPM = reads per million.

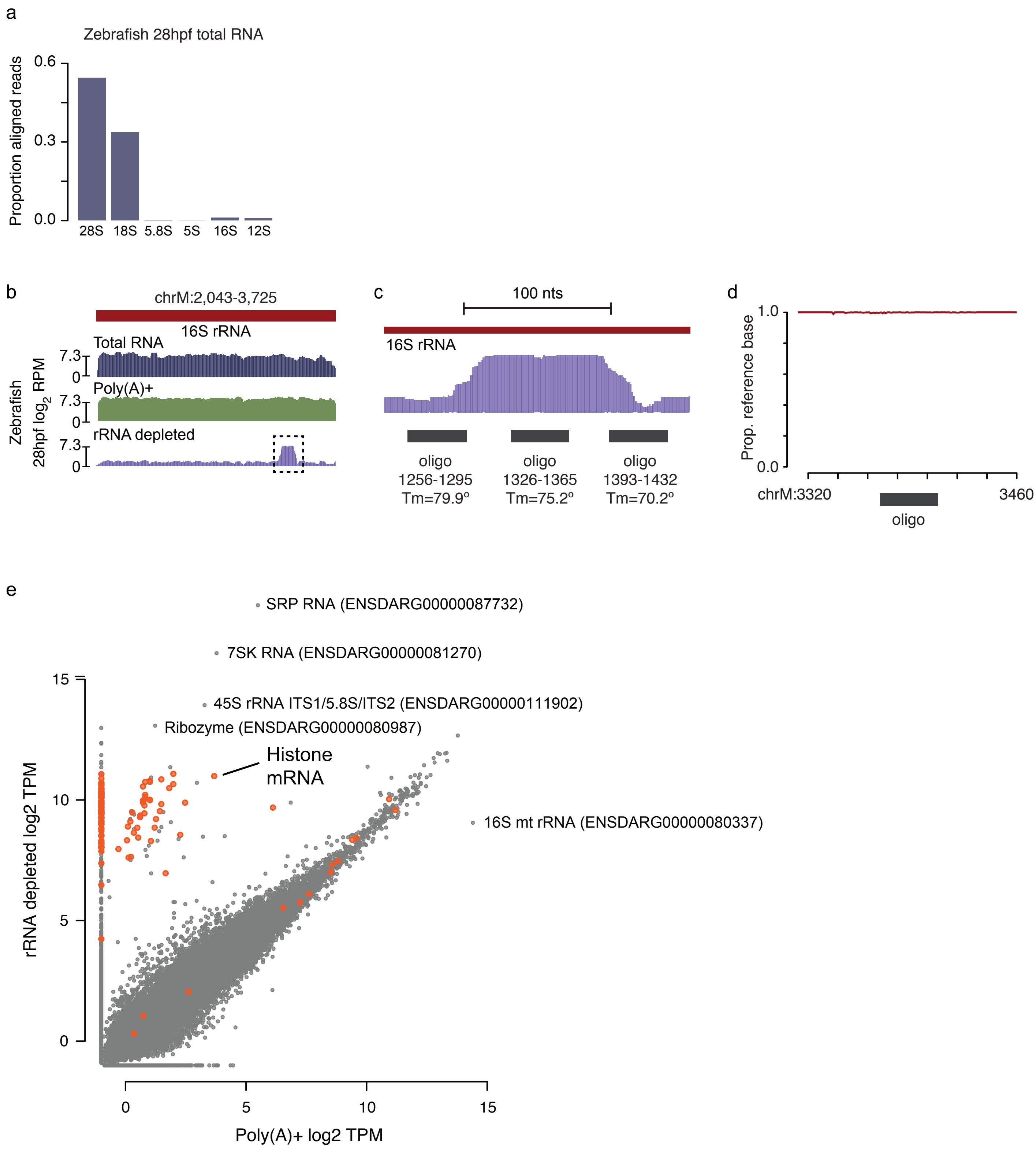

Supplementary Figure S3

Supplementary Figure S3: a) Barplots showing proportion of aligned reads from untreated total RNA mapping to rRNA species in zebrafish. b) Genome browser tracks comparing read coverage at the 16S rDNA loci in untreated, poly(A)+ and rRNA depleted libraries from zebrafish 28 h.p.f. embryos. One region with less efficient rRNA depletion is boxed. c) Zoomed browser track for the boxed region in (b) showing that the inefficient digestion occurred over the region targeted by one oligo (positions 1326-1365 relative to the 16S sequence). This likely resulted in rRNA fragments that were slightly too large to be efficiently excluded during cleanup and library build. d) Variant analysis of sequencing reads mapping to chrM:3320-2460 showing that nearly all read sequences match the GRCz11 reference sequence, suggesting that there is no defect in the oligo's ability to target; rather, it is likely that this oligo was omitted from the pool in error. e) Biplot comparing log2 TPM expression levels from poly(A)+ and rRNA-depleted libraries at 28 h.p.f. Histone genes are highlighted in orange. Several highly expressed non-coding RNAs are labeled. h.p.f. = hours post fertilization, RPM = reads per million, Tm = melting temperature, TPM = transcripts per million.

103 oligos ◯ Oligo list ◉ Full details ⓘ

Export

Name	Target	Start	End	Length	L_gap	R_gap	Tm	GC	Wcards	Expands	N_targets	Antisense_oligo
oligo_001	28S_maternal	25	64	40	24	27	67.8	0.4	0	1	2	TCTTTTCCTCCGCTTAGTAATATGCTTA
oligo_002	28S_maternal	92	130	39	27	20	79.6	0.67	0	1	1	GGGCGGGGATTCGGCGCTGGACTTTTCC
oligo_003	28S_maternal	151	189	39	20	14	81.5	0.79	0	1	1	CGGCCCGCGCCGAGAGAGGCGGCCCTCC
oligo_004	28S_maternal	204	243	40	14	31	78.3	0.64	1	2	2	ACCGGCCTCACACCGTCCRCGGGCTAAG
oligo_005	28S_maternal	275	313	39	31	6	73.8	0.62	0	1	2	CCGCTTTGGGCTGCATTCCCAAACAACC
oligo_006	28S_maternal	320	358	39	6	29	73	0.49	0	1	2	CTATCGGTCTCGTGCCGGTATTTAGCCT
oligo_007	28S_maternal	388	426	39	29	26	70.7	0.46	0	1	2	ACGGTTTCACGCCCTGTTGAACTCTCTC
oligo_008	28S_maternal	453	491	39	26	25	80	0.82	0	1	1	CGACCCCGCCCCGCCGGGCTGAATCCAC
oligo_009	28S_maternal	517	556	40	25	29	80	0.75	0	1	1	CGGGCGTCAACCCCCCGCGCCACCCCCA
oligo_010	28S_maternal	586	625	40	29	8	79.2	0.8	0	1	1	GCCGAACCGGAGCCGGTCGCGGCGCTCC
oligo_011	28S_maternal	634	672	39	8	0	78.5	0.74	0	1	1	CCGACGGCCTGAACCGACGGGCCACCTT
oligo_012	28S_maternal	673	711	39	0	17	74.6	0.71	1	2	2	AGCAAGCGGCGAAGTCGGGGCGGGRGGG
oligo_013	28S_maternal	729	767	39	17	28	79.6	0.72	0	1	1	CCCACAGGGAGGGAGGGCAGAGAGGGCG
oligo_014	28S_maternal	796	834	39	28	30	80.1	0.74	0	1	1	CGGACTGAGGACAGTCCACCCCTGTCGC
oligo_015	28S_maternal	865	904	40	30	30	80.5	0.78	0	1	1	GTCGCCGCGGACCTCGGGCGCCCGAACG

Supplementary Figure S4: Oligo-ASST Web screenshot of the detailed results from designing antisense oligos targeting the zebrafish maternal and somatic 28S rRNAs together.

Supplementary Figure S4

Supplementary File: Protocol for RNaseH-mediated RNA depletion

Optimized design of antisense oligomers for targeted rRNA depletion

Wesley A. Phelps, Anne E. Carlson, Miler T. Lee

Overview:

This protocol describes how to deplete abundant RNA species (e.g., ribosomal RNA) from total RNA using the method described in Phelps et al, 2020. Antisense DNA oligos that sparsely tile target RNA sequences are designed using Oligo-ASST, https://mtleelab.pitt.edu/oligo , and used in an RNaseH digestion reaction followed by column clean up. Depleted RNA is suitable for downstream applications such as RNA-seq and qRT-PCR.

Materials:

Equipment:
Thermal cycler
Centrifuge
Pipets to measure 1 to 700 µL

Consumables:
PCR tubes and caps compatible with the thermal cycler
Aerosol filter pipet tips
1.5 mL tubes

Reagents:
5x hybridization buffer (500mM Tris-HCl pH 7.4, 1M NaCl, 50mM DTT)
Nuclease-free water
100% ethanol
RNase-Away decontamination reagent (Invitrogen #10328011)
Zymo Clean and Concentrator-5 kit (#D4013)
NEB Thermostable RNaseH enzyme and 10x buffer (#M0523S)
TURBO DNase enzyme and 10x buffer (Invitrogen #AM2238)

Custom reagents:
Antisense DNA oligos targeting RNAs to be depleted (e.g., rRNAs)
Purified total RNA

Method:

Oligo design (once per target RNA)

1. Curate the target RNA sequences using Web databases such as Genbank, the UCSC Genome Browser, or Ensembl. For eukaryotic rRNA depletion, you would typically include the three nuclear rDNA sequences (e.g., 28S, 18S and 5.8S) and the two mitochondrial rDNA sequences (16S and 12S).

2. Design antisense oligos using the Oligo-ASST Web tool, https://mtleelab.pitt.edu/oligo . Input your sequences one at a time or all at once in a single FASTA file and click the “Calculate” button to use the default settings (39-40 nt oligos spaced ≤30 nts apart, with melting temperature as

close to 70-80ºC as possible). Download the oligo sequences to your computer using the “Export” button and open the file using Microsoft Excel or similar program.

3. Order the oligos at the desired scale and standard desalting – 25 nmol scale per oligo should be sufficient for >1000 nuclear rRNA depletion reactions from 1µg of total RNA. Some vendors offer bulk/value pricing when ordering above a threshold number of oligos. (Warning: if you downloaded the Details file from Oligo-ASST, be sure to order the antisense oligos and *NOT* the target sequences).

All remaining steps should be done in conditions that minimize contamination with unwanted RNases. Wipe all surfaces and tools with RNase-Away and use filter tips and clean consumables.

Constructing oligo pools

4. Resuspend each dry oligo tube to 1000 µM with nuclease-free water.

5. Create a 10x working stock of oligos by combining 1µL of each oligo into a tube, then diluting with nuclease-free water to the desired concentration per oligo. For eukaryotes, a nuclear rRNA 10x stock should be 4 µM per individual oligo, to be about 10-fold in excess of the target RNA species in 1µg of total RNA in a 10 µL hybridization reaction. A 10x stock at 1µM per oligo may be sufficient for a mitochondrial rRNA pool, depending on taxon and cellular context.

Oligo hybridization to RNA

6. Combine 1 µL of each 10x oligo pool (e.g., nuclear rRNA pool + mitochondrial rRNA pool) with 1 µg of total RNA in a PCR tube.

7. Add 2 µL of 5x hybridization buffer and bring the reaction to 10 µL with nuclease-free water.

8. Mix well by pipetting and place the capped tube in the thermocycler with the following hybridization program:

    a. 95°C for 2 minutes (this facilitates RNA denaturation)

    b. Cool the reaction to 22°C at a rate of 0.1°C/s and hold at 22°C for 5 minutes

9. Place the reaction on ice.

RNaseH-mediated depletion

10. Add 10U of thermostable RNaseH and 2µL of 10x RNaseH buffer to the reaction. Bring the reaction volume to 20µL with nuclease-free water.

11. Mix well by pipetting and heat the depletion reaction at 65°C for 5 minutes or 45ºC for 30 minutes. Reaction temperature should be near or above the predicted melting temperatures of the oligos.

12. Place the reaction on ice.

13. Add 5U of TURBO DNase and 5µL of 10x DNase buffer. Bring the reaction volume to 50µL with nuclease-free water.

14. Mix well by pipetting and heat the reaction at 37°C for 30 minutes.

15. Place the reaction on ice.

Clean-up and size selection

Follow the manufacturer's protocol for the Zymo Clean and Concentrator-5 kit to enrich for RNA molecules >200 nts:

16. Mix 50 µL of Zymo RNA Binding Buffer and 50 µL of 100% ethanol to create the Adjusted Binding Buffer.

17. Add 100 µL (2 volumes) of Adjusted Binding Buffer to depleted sample, which should be in a volume of 50µL after Step 15. Mix well by pipetting.

18. Transfer the sample to a Zymo spin column with collection tube. Centrifuge for 30 seconds at 15,000 x g.

19. Discard the flow through, which will contain RNAs < 200 nts

20. Add 400 µL of RNA Prep Buffer to the column. Centrifuge for 30 seconds at 15,000 x g. Discard flow through.

21. Add 700 µL of RNA Wash Buffer to the column. Centrifuge for 30 seconds at 15,000 x g. Discard flow through.

22. Add 400 µL of RNA Wash Buffer to the column. Centrifuge for 1 minute at 15,000 x g. Discard flow through.

23. Place the column into a nuclease-free 1.5 mL microcentrifuge tube for elution.

24. Add 6 µL of nuclease-free water directly to the column matrix and centrifuge for 30 seconds at 15,000 x g.

25. Proceed straight to downstream application (e.g., RNA-seq library building) or store purified RNA at -80°C until needed.
